# Pharmacological activation of PIEZO1 in human red blood cells prevents *Plasmodium falciparum* invasion

**DOI:** 10.1007/s00018-023-04773-0

**Published:** 2023-04-18

**Authors:** Rakhee Lohia, Benoit Allegrini, Laurence Berry, Hélène Guizouarn, Rachel Cerdan, Manouk Abkarian, Dominique Douguet, Eric Honoré, Kai Wengelnik

**Affiliations:** 1 LPHI, University of Montpellier, CNRS UMR5294, Montpellier, France; 2 iBV, Université Côte d’Azur, CNRS, INSERM, Nice, France; 3 Centre de Biologie Structurale, CNRS UMR5048, INSERM U1054, University of Montpellier, Montpellier, France; 4 IPMC, University Côte d’Azur, CNRS, INSERM, UMR7275, Labex ICST, Valbonne, France; 5 LPHI, University of Montpellier, CNRS UMR5294, INSERM, Montpellier, France

**Keywords:** Erythrocyte, Mechanosensitive ion channel, Piezo1, Malaria, *Plasmodium falciparum*

## Abstract

An inherited gain-of-function variant (E756del) in the mechanosensitive cationic channel *PIEZO1* was shown to confer a significant protection against severe malaria. Here, we demonstrate in vitro that human red blood cell (RBC) infection by *Plasmodium falciparum* is prevented by the pharmacological activation of PIEZO1. Yoda1 causes an increase in intracellular calcium associated with rapid echinocytosis that inhibits RBC invasion, without affecting parasite intraerythrocytic growth, division or egress. Notably, Yoda1 treatment significantly decreases merozoite attachment and subsequent RBC deformation. Intracellular Na^+^/K^+^ imbalance is unrelated to the mechanism of protection, although delayed RBC dehydration observed in the standard parasite culture medium RPMI/albumax further enhances the resistance to malaria conferred by Yoda1. The chemically unrelated Jedi2 PIEZO1 activator similarly causes echinocytosis and RBC dehydration associated with resistance to malaria invasion. Spiky outward membrane projections are anticipated to reduce the effective surface area required for both merozoite attachment and internalization upon pharmacological activation of PIEZO1. Globally, our findings indicate that the loss of the typical biconcave discoid shape of RBCs, together with an altered optimal surface to volume ratio, induced by PIEZO1 pharmacological activation prevent efficient *P. falciparum* invasion.

**Supplementary Information:**

The online version contains supplementary material available at 10.1007/s00018-023-04773-0.

## Introduction

Malaria is caused by the cyclic infection and destruction of red blood cells (RBCs) by a protozoan parasite of the genus *Plasmodium*, the most deadly form being caused by *P. falciparum*. There is substantial variability in susceptibility to malaria in the human population and, in numerous cases this has been attributed to particular features of human RBCs. Sickle cell anaemia, caused by a point mutation in haemoglobin (Hb) leading to the production of HbS, is the most well-known condition conferring resistance to malaria [1]. Although having a drastic impact on well-being and survival, there is still continuous selection for these mutations in malaria endemic countries [2]. In addition, many other variations in RBC proteins and physiology were suggested to be linked to reduced susceptibility to malaria, including RBC dehydration caused by a variety of clinical or experimental conditions [3, 4].

PIEZO1 and PIEZO2 are mechanosensitive non-selective cationic channels, permeable to Na^+^, K^+^ and Ca^2+^ [5]. PIEZO1 is activated by a variety of mechanical stress, including local membrane stretch, whole cell indentation or shear stress [6]. PIEZO1 and PIEZO2 homologs, which share 43% amino acid sequence identity, are conserved during evolution from plants, worm, fly, fish to human [5], while no orthologue is present in *Plasmodium* species or other apicomplexan parasites [7]. PIEZO1 and PIEZO2 are differentially expressed across different tissues and cell types, with only PIEZO1 present in RBCs [5, 8]. PIEZO1 and PIEZO2 are made of a remarkably large homotrimeric complex (about 900 kDa, including 114 transmembrane segments) with a curved shape, similar to a three blades propeller (or a triskelion) [9–13]. PIEZO1 curves the membrane, forming an inverted cone that reversibly flattens upon activation in response to mechanical stimulation [10, 14, 15].

Gain-of-function (GOF) *PIEZO1* mutations causing a delayed inactivation (i.e., prolonged opening), are linked to hereditary xerocytosis, a mild haemolytic anaemia associated with RBC dehydration [16–19]. Remarkably, mice carrying a xerocytosis mutation (mR2482H) either in all hematopoietic cells or specifically in erythrocytes show RBC dehydration and are protected from cerebral malaria caused by the rodent-specific parasite *Plasmodium berghei* [20, 21]. *Piezo1* R2482H mice show no damage of the blood–brain barrier, while wild type mice succumb to cerebral malaria leading to death within 5–7 days post infection. Along this line, another GOF *PIEZO1* variant (E756del), present in about 1/3 of the black population from Western Africa, also confers a significant resistance against severe malaria [20, 21]. Recently, it was confirmed that in children from Gabon, the *PIEZO1* E756del variant is strongly associated with protection against severe malaria in heterozygotes, independent of the protection conferred by the sickle cell trait (HbS) [21]. However, another report found no significant protection of the *PIEZO1* E756del variant against severe malaria in a Ghanaian study [22]. Whether or not the protective effect of *PIEZO1* E756del is linked to RBC dehydration and a decreased intracellular growth of *P. falciparum* is also disputed [20, 21, 23]. Moreover, additional evidence indicates that the beneficial effect of *PIEZO1* R2482H is not solely due to an effect on RBCs, but also involves the immune system [20]. *PIEZO1* E756del has also been shown to lead to iron overload by increasing RBC turnover through phagocytosis by macrophages, although whether or not this effect may be related to malaria protection is unknown [20, 24]. All together these data indicate that PIEZO1 GOF is protective against severe malaria, although the mechanisms implicated still remain unclear and controversial [20, 21, 23].

The pharmacology of PIEZO1 is still at its early ages. Yoda1 was identified by a high throughput screening of small molecules as an activator of mouse and human PIEZO1, without affecting PIEZO2 [25]. When analyzed in HEK 293T cells expressing either mouse or human PIEZO1, Yoda1 (in the micromolar range) causes enhanced opening of PIEZO1 in response to mechanical stimulation, together with a delayed inactivation [25]. Yoda1 acts as an activator of PIEZO1 (not as a true opener) by shifting its pressure-effect curve towards lower pressure values [25]. Two additional compounds Jedi1 and Jedi2 (chemically unrelated to Yoda1) similarly activate PIEZO1, although at a concentration in the millimolar range [26].

In mouse RBCs, Yoda1 causes a robust increase in intracellular calcium, without the requirement of a mechanical stimulation [8]. Notably, addition of 15 µM Yoda1 to RBCs leads to a dramatic change in cell shape, inducing echinocytosis within 30 s [8]. This change in cell shape is strictly dependent on the presence of Piezo1 since RBCs from knock out mice are not altered by Yoda1 [8]. Yoda1 induces mouse RBC dehydration through the secondary activation of the Ca^2+^-dependent Gardos (KCNN4/KCa3.1) channel and consequent water loss by aquaporins [8, 27].

Although the link between inherited *PIEZO1* GOF variants and resistance against malaria is now well established, the mechanism(s) of protection still remain elusive and controversial [20, 21, 23]. In this study, taking advantage of an in vitro model of human RBC infection by *P. falciparum*, we aimed at exploring the antimalarial activity of PIEZO1 pharmacological activators and at determining which step of the infection cycle is PIEZO1 sensitive. Altogether, our findings show that the in vitro pharmacological activation of PIEZO1 in human RBCs prevents parasite invasion. PIEZO1 activation by Yoda1 or Jedi2 deforms the RBC membrane (echinocytosis) causing a loss of typical RBC discoid shape, anticipated to prevent merozoite attachment and internalization. Secondarily, RBC dehydration (that is delayed in the standard RPMI/albumax parasite culture medium) further contributes to enhance protection against malaria, as previously observed for the *PIEZO1* E756del GOF variant [4, 20, 21, 23]. Thus, our findings indicate that both membrane deformation and RBC dehydration contribute to decrease efficient *P. falciparum* invasion.

## Results

### The PIEZO1 activator Yoda1 increases intracellular calcium and causes transient echinocyte formation in human RBCs

**Fig. 1:**
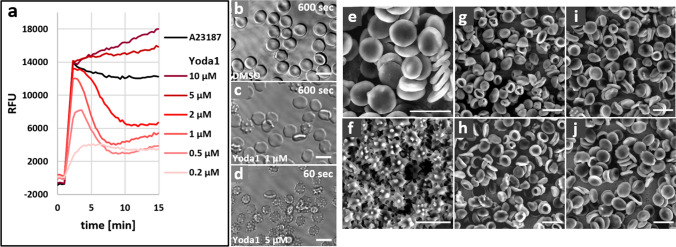
Echinocytic effect of the PIEZO1 activator Yoda1 on human RBCs.** a** Kinetic analysis of the calcium signal elicited by the addition of Yoda1 at the indicated concentrations in RPMI medium. RBCs were loaded with Fluo4-AM and the calcium ionophore A23187 (2 µM) was used as positive control. Background readings of DMSO control samples were subtracted (*n* = 4). **b** Morphological changes of RBCs over 10 min upon addition of 1 µM and 5 µM Yoda1. Stills of video microscopy (shown in supplemental data) for DMSO control (**b**, video 1) and 1 µM Yoda1 (**c**, video 2) after 10 min, and after 1 min for 5 µM Yoda1 (**d**, video 3). **e**–**j** Scanning electron microscopy images of RBCs. **e** DMSO mock treated, **f** in the presence of 5 µM Yoda1 after 10 min, **g**–**j** after treatment with 5 µM Yoda1 for 10 min, followed by washes and incubation in complete medium at 37 °C for 30 min (**g**), 2 h (**h**), 6 h (**i**), and 24 h (**j**)

We evaluated in vitro the effect of PIEZO1 activation by Yoda1 on human RBC infection by the malaria parasite *Plasmodium falciparum*. Parasite cultures are classically performed in a complex medium consisting of RPMI 1640 supplemented with albumax, allowing an optimal infection and host/parasite survival [28–30]. We first monitored intracellular calcium in Fluo4-AM loaded human RBCs in response to increasing concentrations of the PIEZO1 activator Yoda1 in the RPMI culture medium [8]. The calcium response was biphasic at lower Yoda1 concentrations with an early transient component followed by a plateau, while maintained at higher concentrations similar to the calcium ionophore A23187 response (Fig. 1a). Taking advantage of video microscopy, we examined the change in RBC cell shape upon Yoda1 addition at increasing concentration of Yoda1. Human RBCs became echinocytic in a dose-dependent manner and within less than 30 s with 5 μM Yoda1 (Fig. 1b–d, Videos 1, 2, 3). We also followed the development of RBC morphology over more prolonged periods of time (up to 3 h) in the presence of Yoda1. Video-microscopy revealed that Yoda1 treated RBCs changed their shape over time from echinocytes to rounded cells (discocytes or stomatocytes) within about 2 h at all concentrations tested (Supplemental Fig. 1, Video 4).

Scanning electron microscopy images of RBC in the presence of 5 µM Yoda1 for 10 min further examined in detail the changes in cell shape (Fig. 1e, f). RBC readily changed to echinocytes in the presence of Yoda1 characterized by many tiny outward spikes. These changes were fully reversible upon wash out of Yoda1 (Fig. 1g–j).

Thus, our findings indicate that in the standard parasite RPMI culture medium (only containing 0.42 mM calcium), Yoda1 causes an increase in intracellular calcium associated with transient echinocyte formation. Notably, prolonged incubations of RBC cultures with Yoda1 did not induce cell lysis, with the exception of a mild haemolytic activity detected at the highest concentration tested (10 µM) after 24 h incubation (Supplemental Fig. 2). Accordingly, we used concentrations of Yoda1 ranging from 1 to 5 µM for RBC infection experiments described below.

### Yoda1 induces delayed human RBC dehydration in the standard RPMI/albumax parasite culture medium

**Fig. 2:**
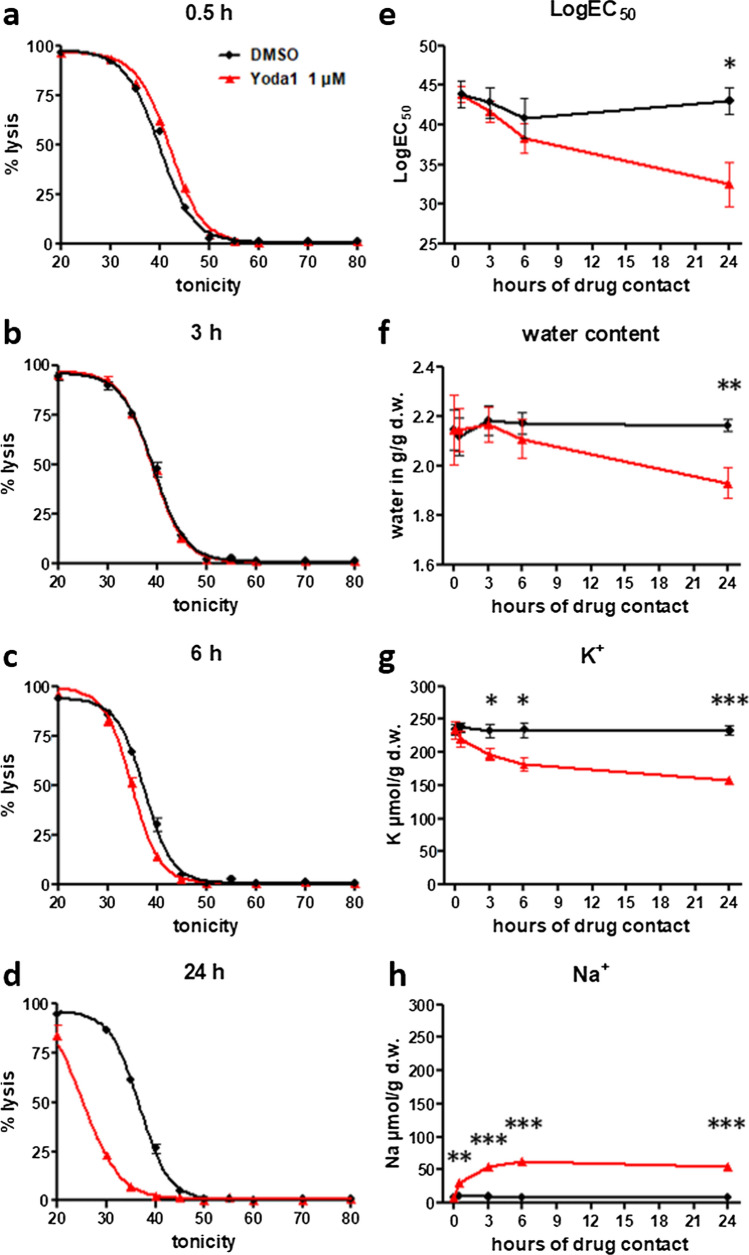
The hydration- and cation status of Yoda1 treated human RBCs over time. **a–d** Osmotic fragility assays were performed on human RBCs that had been treated for the indicated times (0.5–24 h) at 37 °C in complete medium RPMI/albumax with 1 µM Yoda1 or DMSO vehicle (*n* = 4). **e** Summary of the RBC hydration status over time. The LogEC_50_ values were calculated form the curves in **a**–**d**. Shown are mean ± SEM, *n* = 4. **f** Determination of the hydration status over time of treatment in RPMI/albumax (*n* = 3). Intracellular K^+^ (**g**) and Na^+^ content (**h**) for RBC cultures that had been incubated for the indicated time in the presence of 1 µM Yoda1 or DMSO vehicle, *n* = 3. Graph **e** to **h** show the mean ± SEM. *p < 0.05, **p < 0.01, ***p < 0.0001

Next, we investigated whether RBC dehydration occurs in the RPMI medium, as previously reported for mouse RBCs cultured in a saline solution containing 2 mM calcium [8]. We used osmotic fragility assays to quantify RBC dehydration [8]. A leftward shift of the osmotic fragility curve indicates RBC dehydration. RBCs were cultured in the standard RPMI/albumax culture medium (containing 0.42 mM calcium) for up to 24 h with 1 µM Yoda1 to determine their osmotic fragility over time (Fig. 2a–e). Dehydration was observed in the presence of 1 µM Yoda1, but only after incubations of 6 h or longer. These results were confirmed using an independent assay based on the determination of intracellular water content (Fig. 2f). For comparison, when RBCs were incubated in a saline solution containing 2 mM calcium [8], significant dehydration was already observed for a 30 min long incubation with 1 μM Yoda1 (Supplemental Fig. 3).

In addition, we analyzed the intracellular sodium and potassium content of human RBCs in RPMI/albumax treated with 1 µM Yoda1 over a period of 24 h (Fig. 2g, h). At 1 µM Yoda1 an excess loss of K^+^ with respect to a gain in Na^+^ was consistently observed along with progressive dehydration over time.

Thus, in RPMI a progressive change in Na^+^ and K^+^ content associated with delayed RBC dehydration is observed in the presence of 1 µM Yoda1 after an incubation period for more than 6 h, probably related to the low extracellular calcium concentration (0.42 mM) of the RPMI/albumax medium.

### Low dose of Yoda1 prevents RBC invasion by *P. falciparum* merozoites

**Fig. 3:**
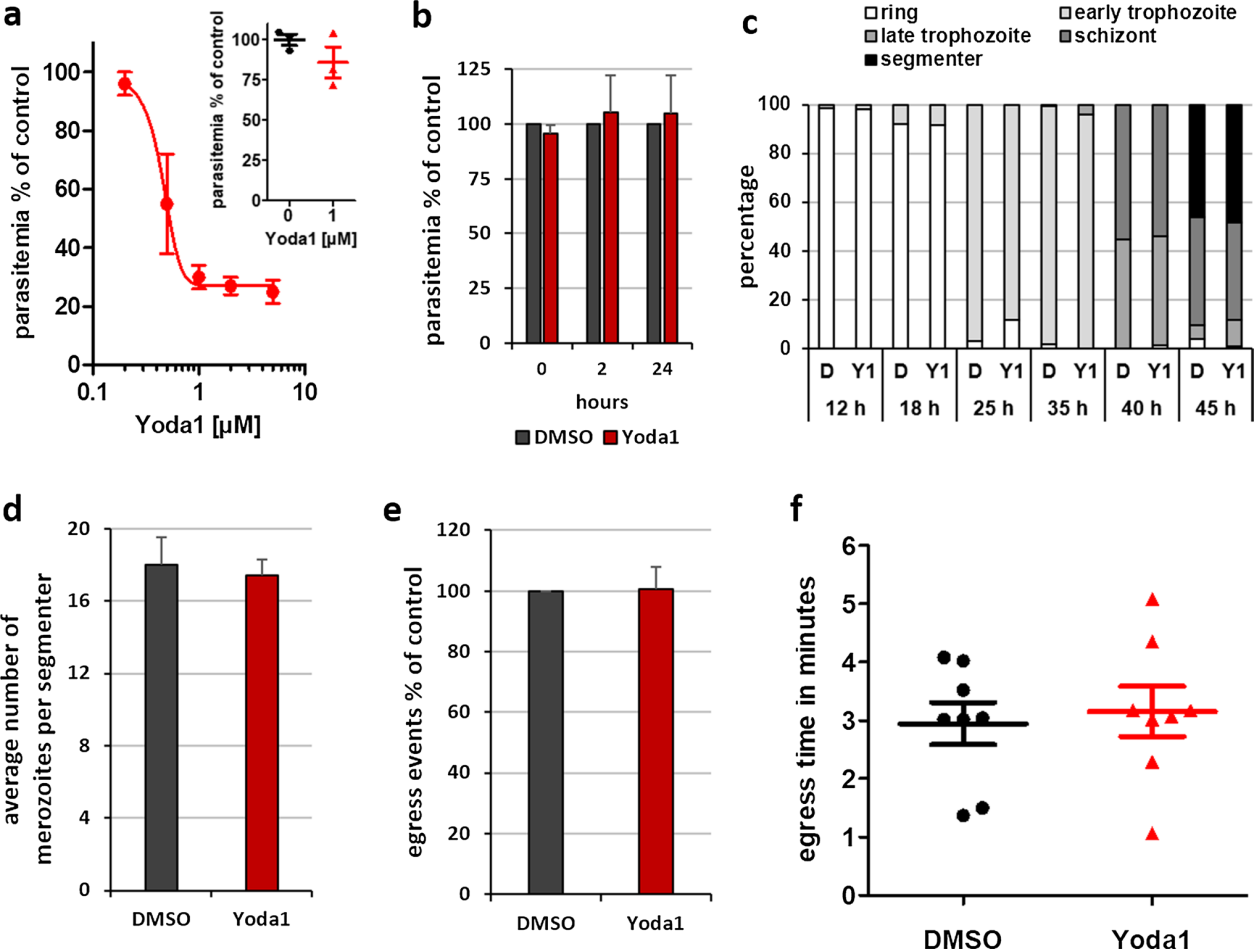
Pharmacological activation of PIEZO1 reduces *P. falciparum* parasitemia but does not affect intra-erythrocytic growth and development in vitro. **a** Synchronized ring stage parasite cultures in RPMI/albumax were incubated for 60 h in the continuous presence of Yoda1 at different concentrations before determining the parasitemia by FACS analysis (*n* = 5). The inset shows the reversibility of inhibition with 1 µM Yoda1. Uninfected RBCs were first pre-treated in RPMI/albumax with 1 µM Yoda1 for 10 min and then washed extensively before adding purified schizont/segmenter stage infected RBCs for culture in complete medium. Parasitemia was determined after incubation for 18 h (*n* = 3). **b** Yoda1 (5 µM) was added to ring stage parasites (0–4 h post invasion) and parasitemia determined after 2 and 24 h incubation (*n* = 3). **c** The progression through the intra-erythrocytic development in the presence of 5 µM Yoda1 was followed by Diff-Quick-stained thin smears. At 40 h, cultures were treated with PKG inhibitor C2 to block egress (*n* = 3). **d** The number of merozoites formed per segmenter was counted on stained thin smears after 45 h culture in the presence of 5 µM Yoda1. Egress had been blocked by C2 treatment (*n* = 2). **e** Relative number and **f** timing of egress events were determined from video microscopy analysis. Purified segmenter-stage parasites had been blocked before egress with C2. Upon removal of inhibitor by a rapid wash, parasites were added to RPMI/albumax containing 5 µM Yoda1 or DMSO vehicle and monitored in parallel allowing comparison of the total number of egress events and the relative time to first egress (after start of the video acquisition) between the two conditions (*n* = 2).** a**–**f** Shown are mean values and SEM

We investigated whether or not chemical activation of PIEZO1 with Yoda1 may influence *Plasmodium falciparum* infection of human RBCs using the standard parasite culture medium. Synchronized ring stage parasites were incubated for 60 h in the presence of different concentrations of Yoda1. All assays with parasites were performed in the standard RPMI/albumax parasite culture medium [29]. Notably, a dose-dependent decrease in parasitemia was observed with an IC_50_ value of about 500 nM Yoda1 (Fig. 3a). The protective effect of 1 µM Yoda1 was fully reversible upon wash, indicating that the protection against infection was not due to RBC damage (Fig. 3a, inset).

The observed reduction in parasitemia caused by Yoda1 could be due to selective lysis of infected RBCs, reduced growth and development of parasites, interference with parasite egress from the infected RBC, reduction of RBC invasion, or to a combination of these effects. We observed no reduction in parasitemia when synchronised ring stage parasites were incubated with Yoda1 for 24 h (Fig. 3b), arguing against Yoda1-induced selective lysis of infected RBCs. Moreover, progression through the different stages of parasite maturation was not affected in the presence of Yoda1 (Fig. 3c). The same number of daughter cells were formed in the presence or absence of Yoda1 (Fig. 3d). Altogether, these results show that intracellular parasite development is not affected by Yoda1, indicating that either parasite egress or RBC invasion was affected.

We observed parasite egress by live microscopy in the presence of Yoda1. Highly synchronised late-stage parasite-infected RBCs were purified and allowed to mature to the segmenter stage (when daughter merozoites are fully formed and are close to egress) in the presence of the protein kinase G (PKG) inhibitor C2; this treatment reversibly blocks parasite egress from the host RBC [31]. The block of egress was then lifted in the presence of Yoda1 or of vehicle and both samples were observed in parallel by live microscopy. Counting the number of egress events (Fig. 3e) and determining the relative time of the first egress event revealed that there was no difference between the two samples (Fig. 3f). Thus, Yoda1 inhibits invasion of human RBCs by *P. falciparum*, but does not interfere with parasite growth or egress.

### Yoda1 impairs parasite attachment to the RBC membrane

**Fig. 4:**
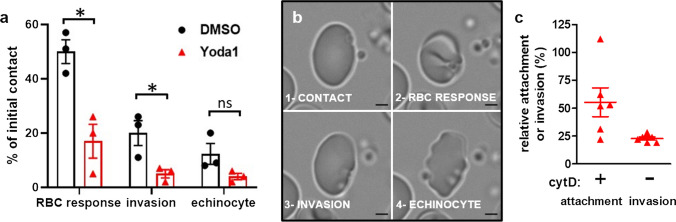
Effect of Yoda1 on merozoite attachment and invasion. **a** Quantification of invasion events in the presence and absence of Yoda1. Highly synchronous purified parasites were mixed with RBC preparations that had been pre-treated for 10 min with 5 µM Yoda1 or DMSO 5 h prior to the experiment. Events subsequent to egress were observed by video microscopy and classified. Data are expressed with respect to the observed initial possible contact of merozoites with RBCs. Shown are the mean ± SEM of three independent experiments. *p < 0.05, *ns* not significant. **b** Still images of one RBC (DMSO condition, taken from video 5) representing the events that were quantified: merozoite contact, RBC response by deformation, invasion, and echinocyte formation. Scale bar 2 µm. Supplemental video 6 represents an example of the poor reponse of Yoda1-pretreated RBCs upon contact with merozoites. **c** Attachment of merozoites to RBCs pre-treated with 5 µM Yoda1 was quantified in the presence of 1 µM cytD and expressed as percentage of mock-treated control RBCs. In the presence of Yoda1 and in the absence of cytD we observed the reduction in parasitemia as described in Fig. 3A (*n* = 6, mean ± SEM, *p* < 0.0001 for both conditions)

To analyze in closer detail which step in parasite invasion is impaired by Yoda1 we explored the initial cellular events occurring upon invasion of control and Yoda1-treated RBCs by *P. falciparum* merozoites using direct observation by video microscopy. Highly synchronized purified segmenter stage parasites were first blocked before egress by treatment with C2. Upon washing off the inhibitor, the infected RBCs were mixed with RBCs that had been pre-treated with Yoda1 or DMSO and then washed. From the analysis of the videos, we quantified the following events that occur in chronological order: (a) the number of RBCs that came close to and likely came in contact with released merozoites; (b) RBCs that deformed upon merozoite contact; (c) invasion events; and (d) echinocyte formation of RBCs as a consequence of completed parasite invasion (Fig. 4a, b, Video 5, Video 6). We observed a major reduction in the number of early RBC deformation, merozoite invasion and echinocyte formation in the presence of Yoda1.

We also determined whether or not the upstream event of merozoite attachment to RBCs would also be modified by Yoda1 treatment. We took advantage of the small molecule inhibitor of actin polymerisation, cytochalasin D (cytD) to allow attachment of merozoites to RBCs, while preventing parasite invasion [32, 33]. In the presence of cytD, pre-treatment of RBCs with Yoda1 followed by washes and recovery for 5 h resulted in a significant decrease in the attachment of the parasite (Fig. 4c). Treatment with heparin that blocks adhesive events likely through interaction with the parasite invasion ligand MSP1 [34, 35], completely abolished attachment and invasion and was used as a control. Thus, invasion by merozoites appears to be blocked at the initial steps of merozoite attachment to the RBC by Yoda1.

### Protection conferred by Yoda1 against *P. falciparum* invasion is independent of an altered intracellular Na^+^/K^+^ balance in human RBCs

**Fig. 5:**
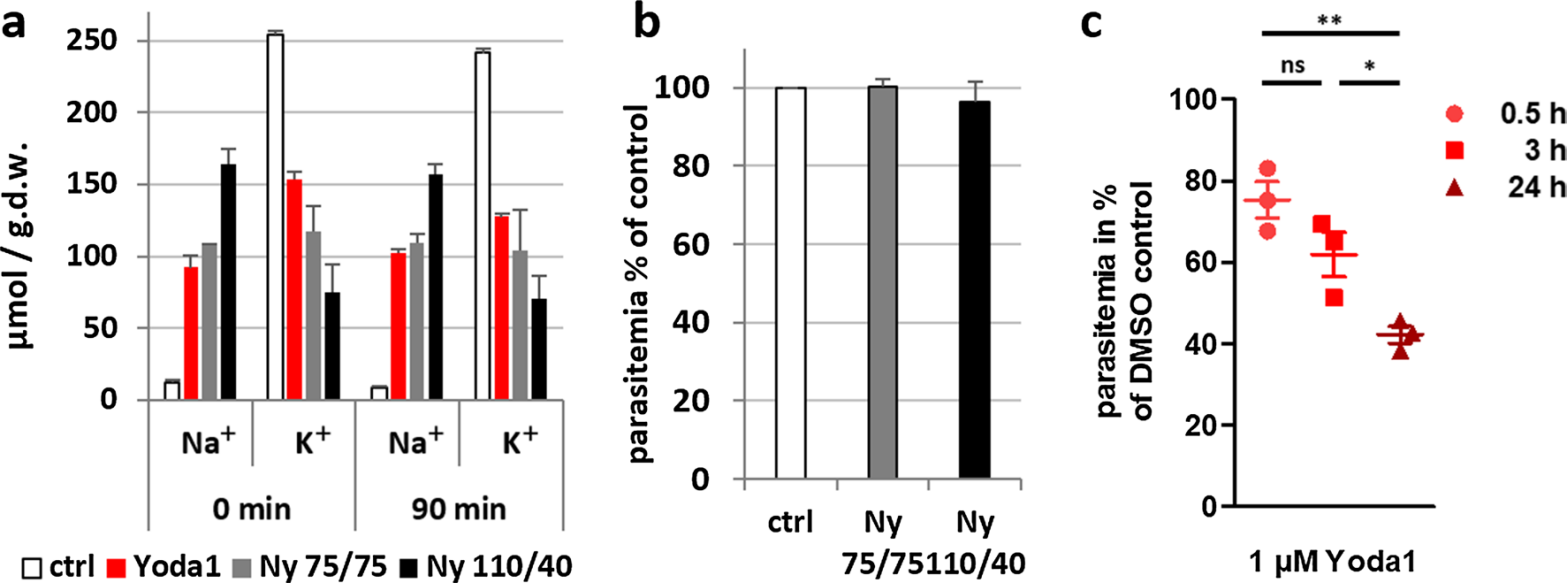
Intracellular Na^+^/K^+^ balance in human RBCs upon Yoda1 treatment and effect on *P. falciparum* infection. **a** Na^+^ and K^+^ content was determined upon standard 10 min treatment with 5 µM Yoda1 either directly after treatment and washes or after further 90 min incubation in RPMI/albumax medium at 37 °C. Treatment of RBCs with the ionophore nystatin in saline solution containing either 75 mM Na^+^ and 75 mM K^+^ (Ny 75/75) or 110 mM Na^+^ and 40 mM K^+^ (Ny 110/40). The resulting intracellular Na^+^ and K^+^ concentrations were determined directly after washing and after a further incubation for 90 min at 37 °C in RPMI. **b** RBCs treated with nystatin in the two saline solutions were used for standard invasion assays with purified highly synchronous late-stage parasites. Results are expressed as percentage of untreated RBC controls (*n* = 3, mean ± SEM). **c** Effect of prolonged treatment with Yoda1 on parasite invasion. RBCs in RPMI/albumax medium were treated with 1 µM Yoda1 for 0.5, 3 or 20 h before purified late stage parasites were added. After 18 h of culture ring-stage parasitemia was determined and expressed as percentage of DMSO-treated controls. Results of three independent experiments performed in duplicates are shown (mean ± SEM). *p < 0.05, **p < 0.01, *ns* not significant

We investigated whether or not changes in intracellular Na^+^ and/or K^+^ concentrations might contribute to the antimalarial properties of Yoda1. We experimentally modified the intracellular Na^+^ and K^+^ content of RBCs by pre-treatment in saline solutions with varying Na^+^/K^+^ concentrations in the presence of the ionophore nystatin (Fig. 5a). In the presence of 75 mM Na^+^/75 mM K^+^ and nystatin, Na^+^ was elevated while K^+^ was decreased, similar to the Yoda1 condition (Fig. 2g, h). This effect was enhanced with the 110 mM Na^+^/40 mM K^+^ and nystatin solution. These changes in intracellular cationic concentration persisted after a washout of 90 min in RPMI (Fig. 5a), i.e., around the time when RBCs would be invaded when cultured in the presence of purified parasites. Importantly, *P. falciparum* infection was not affected by the nystatin treatment with these modified ionic solutions (Fig. 5b), indicating that the antimalarial activity of Yoda1 is not mimicked by experimental conditions that similarly affect the intracellular Na^+^/K^+^ balance.

### RBC dehydration contributes to Yoda1 mediated protection against *P. falciparum* infection

Previous findings indicated that dehydrated RBCs are less susceptible to *Plasmodium* infection [4, 20, 21, 23]. Normal RBCs that had been experimentally dehydrated or RBCs from donors with known hereditary conditions that result in dehydrated RBCs (including sickle cell disease) are greatly protected against *P. falciparum* infection. In the same line, Yoda1, as well as GOF *PIEZO1* mutations (slowing down channel inactivation) cause RBC dehydration in a defined minimal medium and was shown to confer resistance against cerebral malaria [8, 20, 23, 27]. Thus, we investigated to which extent RBC dehydration contributes to the antimalarial activity of Yoda1 in the RPMI/albumax medium [29].

We evaluated whether Yoda1-induced reduction in parasitemia depends on the duration of Yoda1 (1 µM) exposure, previously shown to induce delayed RBC dehydration (Fig. 2e, f). Yoda1 was added to RBC cultures in RPMI/albumax medium either 0.5, 3, or 24 h before starting the experiment by adding purified late stage parasites. Parasitemia was determined as before after 18 h and compared to mock-treated control cells (Fig. 5c).

The reduction in parasitemia initially observed after 30 min Yoda1 treatment of RBCs became significantly more pronounced for longer durations of incubation (Fig. 5c). Thus, the progressive dehydration of RBCs over time upon treatment with 1 µM Yoda1 (Fig. 2e, f) is anticipated to enhance the antimalarial effect of PIEZO1 activation [4, 20, 21, 23]. It should also be noted that RBCs recover a discoid shape after 3–24 h long Yoda1 incubation (Fig. 1e–j). However, in our standard assay conditions, the majority of parasite egress events and subsequent RBC invasion would have taken place within the first 3 h of the experiment, i.e., during the period where we did not measure detectable RBC dehydration. Thus, it is likely that additional mechanisms, besides RBC dehydration, may contribute to the observed reduction in parasitemia upon PIEZO1 activation by Yoda1.

### Antimalarial effect of the PIEZO1 activator Jedi2

**Fig. 6:**
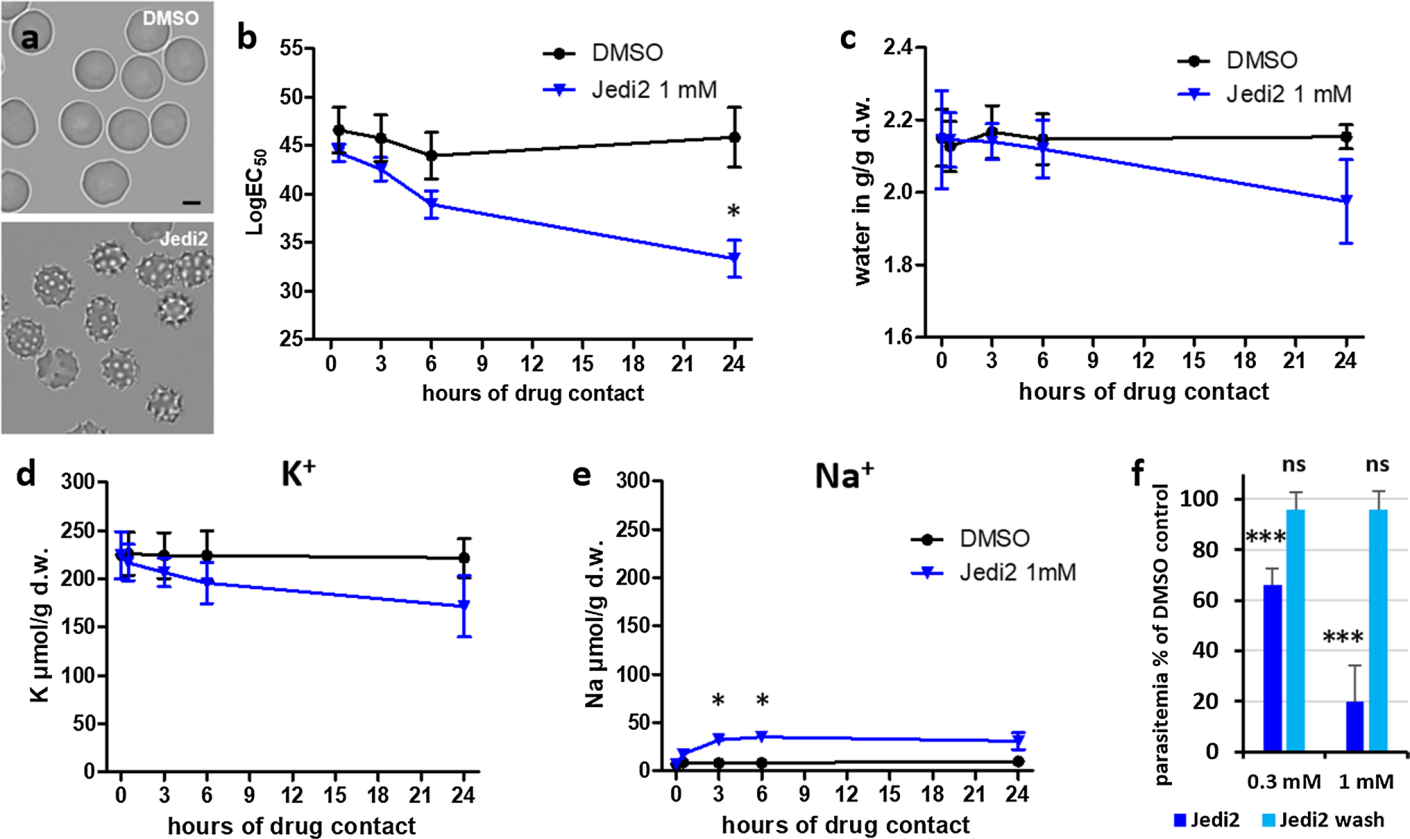
Physiological effects of PIEZO1 activator Jedi2 on human RBCs. **a** Cell shape of the cells at 4 min after addition of compounds (DMSO vehicle and 1 mM Jedi2). Scale bar = 5 µm. **b** Hydration status of RBCs was determined by osmotic fragility assays (Supplemental Fig. 4) after the indicated time of contact of cells with 1 mM Jedi2 in RPMI/albumax medium (*n* = 4). **c** Quantification of hydration status (*n* = 2). **d**, **e** Intracellular K^+^ and Na^+^ concentrations for RBC cultures that had been incubated for the indicated time in the presence of 1 mM Jedi2 in RPMI/albumax (*n* = 2). **f** The effect of Jedi2 on parasitemia was observed either in the continuous presence of Jedi2 in complete medium (RPMI/albumax), or after a pre-treatment for 10 min in RPMI followed by extensive washes (wash) before adding synchronized schizont infected RBCs for culture in complete medium. Parasitemia was determined after incubation for 18 h and expressed as a percentage of mock-treated controls (*n* = 3). All graphs show mean ± SEM. *p < 0.05, ***p < 0.0001, ns or without indication = not significant

We investigated whether or not the antimalarial protection was similarly observed for the PIEZO1 activator Jedi2 that is chemically unrelated to Yoda1 [26]. The small molecule Jedi2 activates PIEZO1 at concentrations in the millimolar range [26]. Our calcium measurements indicated that Jedi2 elicited an increase in intracellular calcium, although weaker than Yoda1 (Supplemental Fig. 4). Similarly to Yoda1, in the presence of Jedi2, RBCs rapidly became echinocytes (Fig. 6a), in the absence of any haemolytic activity (Supplemental Fig. 2). In RPMI culture medium, we observed a time-dependent dehydration of RBCs with 1 mM Jedi2 similar to 1 µM Yoda1 (Figs. 2, 6b, Supplemental Fig. 5). This dehydration was confirmed by measuring RBC water content (Fig. 6c). Jedi2 treatment, similar to Yoda1 (Fig. 2g, h), also affected the intracellular Na^+^ and K^+^ balance (Fig. 6d, e). Again a more important loss of K^+^ with respect to a gain in Na^+^ could explain the observed dehydration after prolonged incubation.

When analyzed on parasite cultures, Jedi2 significantly and reversibly reduced *P. falciparum* parasitemia, similarly to Yoda1 (Fig. 6f, Jedi2). Washing out Jedi2 prior to infection by parasites completely re-established their competence for parasite invasion, as previously observed for 1 µM Yoda1 (Fig. 6f, Jedi wash; Fig. 3a inset).

Altogether, these findings indicate that the chemically unrelated PIEZO1 activators Yoda1 and Jedi2 similarly prevent in vitro parasitemia of human RBCs.

## Discussion

Recent work demonstrates that polymorphisms in the *PIEZO1* gene causing a channel GOF, with a slower inactivation (i.e., increased channel opening), are associated with a protective effect against severe malaria both in humans and in a xerocytosis mouse model [20, 21]. However, the PIEZO1-dependent mechanisms at play conferring the resistance to severe malaria remain debated [20, 21, 23]. Our study focuses on the in vitro pharmacological activation of PIEZO1 in human RBCs and its impact on invasion, growth and development of the malaria parasite *P. falciparum*.

We show that both Yoda1 and Jedi2 PIEZO1 activators [25, 26], have a strong inhibitory effect on parasitemia (in the low micromolar range for Yoda1), reminiscent of the observed reduced multiplication rates in mice carrying *PIEZO1* GOF mutations [20]. Yoda1 does not affect parasite growth or development within RBCs, nor parasite egress from the infected RBCs. However, invasion of RBCs by *Plasmodium* merozoites is potently inhibited by Yoda1. The chemically unrelated PIEZO1 activators Yoda1 and Jedi2, similarly inhibit *P. falciparum* parasitemia (although at different concentrations), suggesting a PIEZO1-dependent mechanism (of note, Piezo1 orthologues are not present in the malaria parasite [7]). Yoda1 induces a major imbalance in intracellular Na^+^ and K^+^ content associated with RBC dehydration, although it is not the primary protection mechanism against malaria. Instead, we provide evidence that Yoda1 inhibits merozoite attachment, in parallel with a dramatic change in RBC shape associated with echinocytosis and formation of numerous spiky membrane projections. Altogether, our in vitro findings further support a role for PIEZO1 in the host RBC contributing to the resistance against severe malaria in vivo conferred by a GOF mutation in PIEZO1 [20, 21]. However, in the case of PIEZO1 GOF variants, echinocytosis was only rarely observed and instead significant RBC dehydration occurred [, 20, 23]. In the present study, RBCs cultured in the standard RPMI/albumax medium only showed significant dehydration after 6 h long drug application, while most of the invasion events were observed to occur within 3 h after the addition of purified *P. falciparum* infected cells to RBCs. Merozoite invasion occurs within minutes of mixing with RBCs in RPMI medium and is fully completed within 30 min at 37 °C [36]. Previous work demonstrated that other molecules causing echinocytosis of human RBCs, including sodium fluoride and phospholipase A2 (presumably unrelated to PIEZO1 activation) similarly prevented *P. falciparum* invasion [37]. These findings, together with our own observations, indicate that loss of the typical discoid shape of RBCs prevents optimal invasion by the malaria parasite.

Sickle cell disease (SCD) has long been recognized to confer a significant protection against malaria [38, 39]. In hypoxic conditions, RBCs from individuals carrying the heterozygote mutation HbS become shaped like sickles or crescent moons. However, RBC invasion is not altered in such conditions [40]. Instead, various alternative mechanisms were implicated in the resistance to malaria in individuals carrying the sickle cell trait, including weakened cytoadherence of infected RBCs, impaired haemoglobin digestion and increased splenic phagocytosis [39, 41, 42]. Thus, both PIEZO1 pharmacological activation (as well as PIEZO1 gain-of-function mutations [20, 21]) and SCD [38, 39] cause a change in the shape of RBCs and confer a significant protection against malaria, although apparently through distinct mechanisms.

Previous studies demonstrated that GOF mutations in *PIEZO1* cause xerocytosis, a mild haemolytic anaemia [16–19]. RBC dehydration was shown in xerocytosis patients [17, 27], in asymptomatic human carriers of a *PIEZO1* E756del polymorphism [20], as well as in an engineered *Piezo1* mouse model of xerocytosis [8, 20]. RBC dehydration arises through PIEZO1 mediated calcium influx leading to activation of the calcium-dependent K^+^ channel Gardos and efflux of K^+^ ions accompanied by water loss via aquaporins [8]. However, our data revealed that under standard *P. falciparum* in vitro culture conditions (in RPMI/albumax, only containing 0.42 mM calcium), RBCs are not significantly dehydrated upon treatment with the PIEZO1 activators Yoda1 or Jedi2 during the first 6 h of drug application. Delayed dehydration in this specific culture medium is likely due to the low extracellular calcium concentration used. Thus, the early protection mediated by PIEZO1 activators against malaria infection (at least in the RPMI medium) does not appear to primarily depend on RBC dehydration. Nevertheless, we observed an increased protection of PIEZO1 opening as a function of the duration of drug application, in parallel with RBC dehydration. Thus, PIEZO1 activation in RBCs by Yoda1 or Jedi2 is anticipated to confer protection against malaria by two independent mechanisms, one involving a loss of discoid shape due to echinocytosis [37] and an additional mechanism involving RBC dehydration [4, 20, 23]. Invasion of RBCs by *P. falciparum* merozoites is a complex process that has mainly been considered from the point of view of the invading parasite (reviewed in [43, 44]). The sequential events for successful invasion are: (1) attachment of the merozoite to the RBC by binding to specific RBC surface proteins triggering waves of RBC deformation; (2) reorientation of the parasite resulting in the apical pole facing the RBC; (3) discharge of apical organelles (micronemes and rhoptries) leading to the formation of a moving junction; (4) invasion by active movement of the parasite using the moving junction as a fixation point for the activity of a parasite actin-myosin motor, leading *in fine* to the formation of a parasitophorous vacuole in which the parasite will develop during its intraerythrocytic growth [45, 46]. How RBC dehydration interferes with parasite invasion remains unclear [4, 20].

The deformation of the RBC during the initial interaction with the merozoite is anticipated to activate PIEZO1. However, no calcium signal in the RBC was reported during the pre-invasion stages (attachment and reorientation) when RBCs maximally deform upon initial merozoite contact (Video 5), suggesting that PIEZO1 activation is unlikely to occur at this stage [35, 47]. Binding of merozoites to human RBCs triggers important biophysical changes that will contribute to facilitate invasion [48]. Modelling has suggested that wrapping of the RBC membrane followed by reorganisation of the underlying cytoskeleton could account for the energetic steps required for the invasion process [49]. Upon contact with the parasite, a signalling pathway through a phosphorylation cascade causes altered viscoelastic properties of the host membrane [50]. A possibility is that these early events may prevent PIEZO1 opening at the very beginning of the invasion process (despite major membrane deformation), thus explaining the absence of a calcium signal at this stage where membrane movements are remarkably large [35, 47]. Moreover, in a quantitative phosphoproteomic analysis of the erythrocyte during the short phase of invasion, PIEZO1 was one of the few identified proteins showing increased phosphorylation upon merozoite attachment prior to invasion [51]. Another question is whether or not the intraerythrocytic parasite may also directly influence PIEZO1 activity within the host cell. Differential phosphorylation of PIEZO1 has been documented in parasitized RBCs [52]. However, the biological significance of PIEZO1 phosphorylation in infected RBCs is unclear at this stage.

Multiple questions remain open to better understand how the pharmacological activation of PIEZO1 confers a potent protection against malaria infection. Recent structural findings indicate that PIEZO1 negatively curves the membrane in the closed state [10, 11, 13, 15]. Upon activation in response to mechanical stimulation, the channel reversibly flattens [15]. How PIEZO1 activation and flattening may influence the shape of RBCs, thereby conditioning merozoite attachment and internalization is an intriguing question. Recent findings indicate that PIEZO1 is preferentially expressed at the RBC biconcave dimple, possibly explained by a curvature coupling between the negative curvature of PIEZO1 at the closed state and the curvature of the RBC membrane [53]. Intuitively, membrane flattening caused by PIEZO1 activation is in opposite direction to membrane internalization during invasion. Moreover, whether or not membrane depolarization (although the resting membrane potential of human RBCs is depolarized in the range of – 10 mV, close to the reversal potential of PIEZO1) and/or the increase in intracellular calcium (although blunted in our standard RPMI medium) are also involved in the mechanism of protection is unknown at this stage. In the same line, PIEZO1 opening in RBCs is anticipated to activate several downstream biochemical cascades (presumably calcium dependent), impacting various structural components of RBCs, including the cytoskeleton [54], as well as the lipid bilayer composition by influencing the cellular lipidome [55]. How these changes may contribute to the protection against malaria infection also needs to be investigated in future studies.

Altogether, our findings indicate that in the case of in vitro pharmacological activation of PIEZO1 by Yoda1 or Jedi2, the key protective event is an early loss of biconcave discoid shape of human RBCs, with secondarily a delayed RBC dehydration.

The *PIEZO1* E756del polymorphism is highly enriched in people of African origin [20]. Remarkably, this variant strongly associates with significant protection against severe malaria, although these heterozygote individuals have no detectable clinical symptoms caused by this deletion [21]. The mechanisms implicated in the protection conferred by the *PIEZO1* E756del against severe malaria appear to be complex and remain disputed [20, 21, 23]. It is likely that a combination of effects involving RBC dehydration [4, 20, 23], decreased expression of the cytoadherence molecule PfEMP-1 [21], and modulation of the immune system [20] mediate the PIEZO1-dependent protection against severe malaria. A recent report further indicates that PIEZO1 E756del opening by either Yoda1 or shear stress produce dehydration-dependent cell haemolysis, thereby inhibiting *P. falciparum* infection [23]. Moreover, 6–13% RBC from PIEZO1 E756del freshly drawn blood show distorted RBCs [23]. In addition, infected PIEZO1 E756del RBCs were also frequently misshaped and distorted. Thus, it is likely that a loss of discoid RBC shape may also contribute to the protection observed in PIEZO1 E756del carriers, although in this case RBC dehydration appears to be a dominant mechanism [20, 21, 23].

Host-directed therapeutic strategies for infectious diseases are becoming viable adjuncts to standard treatments (reviewed in [56]) and were also proposed for malaria [57]. Certain molecules targeting RBC proteins have antimalarial activities [58, 59]. Our in vitro findings now indicate that PIEZO1 pharmacological activation in human RBCs is sufficient to prevent invasion by *P. falciparum*. It is, therefore, tempting to speculate that pharmacological activation of PIEZO1 might constitute an original approach for the development of a future antimalarial treatment, without neglecting the widespread expression of PIEZO1 in human tissues.

## Materials and methods

### Chemicals

The following chemicals and their stock concentrations in DMSO (in square brackets) were used in this study: Yoda1 (Sigma–Aldrich) [20 mM], A23187 (Sigma–Aldrich)[2 mM], Jedi2 (Tocris)[100 mM], Nystatin (Sigma–Aldrich) [40 mg/ml]. The PKG inhibitor C2 was a generous gift of Oliver Billker [3 mM].

### Biological material

Cell cultures of *P. falciparum* 3D7 strain (MRA-102 from MR4, BEI Resources) were performed as previously described [30] in human 0^+^ or A^+^ erythrocytes and complete medium, RPMI 1640 medium with 25 mM HEPES and 2 mM L-glutamine (Gibco), supplemented with 0.5% Albumax I (Gibco), 15 µg/ml hypoxanthine (Sigma–Aldrich) and 40 µg/ml gentamycin (Gibco) at 37 °C under a tri-gaseous mixture of 5% CO_2_, 5% O_2_ and 90% N_2_. Culture conditions were generally static for culture maintenance or with gentle shaking to keep cells in suspension for experiments; this allowed to obtain higher multiplication rates and single infected RBCs. Parasites were synchronized by first purifying late-stage parasites using VarioMACS magnetic cell separator (CS columns, Miltenyi Biotec, Paris, France) [60] or centrifugation on a 63% Percoll/RPMI (GE Healthcare) cushion [61] before culture for 2–4 h with fresh RBCs to allow invasion, followed by a 5% sorbitol treatment to remove remaining late stages [62]. Human blood obtained as donations from anonymized individuals was provided by the local blood bank (Etablissement Français du Sang) under the approval number 21PLER2016-0103. Parasitemia was routinely monitored on thin blood smears fixed in methanol and stained with Diff-Quick™ (pH 7.2) (Dade Behring, France) or by SYBR Green staining and flow cytometry. For microscopic determination of parasitemia, at least 1000 cells were counted. Experiments were generally started with late stage parasites to eventually obtain ring stage parasitemia in the range of 1% and 10%.

### Treatments with Yoda1 and Jedi2

Yoda1 and Jedi2 treatments were performed in two different ways: either in the presence of drug by adding Yoda1/Jedi2 directly to the parasite cultures in complete medium (drug-on experiments) or by pre-treating uninfected RBCs, washing and infection with parasites (drug-off experiments). For the latter, RBCs in RPMI were treated for 10 min at room temperature, washed three times in RPMI, re-suspended in complete medium and mixed with purified infected RBCs that were close to egress. To do so, late-stage parasites from synchronous cultures were purified either by passage over VarioMACS columns or by density centrifugation using a 63% Percoll/RPMI cushion. Pure infected RBCs were washed with complete medium, re-suspended in complete medium with 1.5 µM of the PKG inhibitor C2 [31], gazed and incubated at 37 °C for up to 5 h. This allows parasites to continue their development to segmenter stages but blocks egress until the inhibitor is removed by washing. These parasite preparations were then mixed with either Yoda1/Jedi2 pre-treated RBCs or with mock-treated RBCs. To treat ring stage parasites, purified late stage parasites were mixed with RBCs, incubated for 4 h and synchronized by 5% sorbitol treatment. Cultures were allowed to stabilize for 1 h before adding Yoda1. Individual experiments were generally performed in triplicates and parasitemia determined by FACS. Experiments were repeated at least three times with blood from different donors.

### Parasitemia determination by FACS

Cultures were centrifuged and the cells fixed for at least 4 h at room temperature in 10 volumes of 4% paraformaldehyde (Electron Microscopy Sciences) in phosphate buffered saline (PBS). Preparations were then washed twice with PBS and incubated 30 min with 3.3 × SYBR Green (Thermo Fisher Scientific). DNA dye was removed by a simple wash in PBS and samples analysed on a FACS Canto (BD Biosciences) using processed uninfected RBCs as negative control.

### Prolonged pre-treatment of RBCs with Yoda1 and Jedi2 and inhibition of parasite invasion

Total blood of different donors was washed three times in RPMI. 1 ml cultures at a haematocrit of 5% were prepared in triplicates and treated with drugs or vehicle (DMSO) and incubated in 12 well plates in standard culture conditions at 37 °C with shaking to keep cells in suspension. Treatment was done 24 h, 3 h and 0.5 h prior to the start of the experiment. Synchronised parasites at the schizont stage were purified to more than 90% purity using VarioMACS columns and put back into culture in the presence of 1.5 µM PKG inhibitor C2 for 4 h, what allows parasite development to the segmenter stage but reversibly blocks parasite egress. Parasites were collected by centrifugation at 300 *g* and re-suspended in complete medium. Ten µL suspension were then added per well of the pre-treated RBCs, and cultured under standard condition. After 18 h, 50 µL of culture were transferred to 96-well round bottom plates and fixed with an equal volume of 8% PFA in PBS for 3 h at RT. For FACS analysis, the cells were washed twice with 200 µL PBS and stained with 50 µL 5 × SYBR-Green solution for 30 min in the 96-well plate. Preparations were analyzed on a FACS-CANTO II cytometer (BD Biosciences). 100,000 events were recorded. Ring stage parastemia was determined using FlowJo software (vs10.8, Tree Star).

### Haemolytic activity assays

Blood of different donors was obtained. Throughout these assays RPMI 1640 without phenol red (Gibco) was used. RBCs were washed twice in RPMI and suspensions at 5% haematocrit were prepared in complete medium (RPMI 0.5% albumax) and 100 µL samples were dispatched in round-bottom 96-well culture plates. Yoda1and Jedi2 were prepared in complete medium at double concentration, and 100 µL added to the wells containing 100 µL RBC suspensions in triplicates (2.5% final haematocrit) and mixed by pipetting. Final concentrations for Yoda1 were 10 µM, 5 µM, 2 µM and 1 µM and for Jedi2 1000 µM, 300 µM and 100 µM. Wells with DMSO were prepared for baseline determination (0.1% for Yoda1, 0.5% for Jedi2). Plates were incubated in standard conditions at 37 °C in 5% O_2_, 5% CO_2_. After 24 h, 2 µL of saponin (15%) were added to selected wells to obtain total lysis. RBCs were pelleted by centrifugation for 3 min at 1800* g* and 150 µL of supernatant transferred to flat bottom 96-well plates. Haemoglobin released into the culture supernatant was quantified using a SPARK multifunctional microplate reader (TECAN) at 540 nm. Lysis was calculated after deduction of DMSO treated samples as percentage of total saponin-induced lysis.

### Osmotic fragility assays

Osmotic fragility assays were performed as described [8] with minor modifications [4]. Briefly, blood from different donors was used the day of reception whenever possible. The blood was washed twice either in RPMI or in normal saline (NS, 149 mM NaCl, 2 mM HEPES, pH 7.4) depending on the following experimental conditions. One millilitre blood suspensions at 2.5% haematocrit were incubated with compounds for 30 min at 37 °C in RPMI or in NS supplemented with 4 mM KCl and 2 mM CaCl_2_. Identical quantities of DMSO solvent served as controls. Ten μl of blood suspension were then added to U-bottom 96-well plates. Solutions of varying tonicity were generated by mixing NS (100%) with 2 mM HEPES, pH 7.4 (0%). 250 μl of these solutions were added to the diluted blood and incubated for 5 min at room temperature. Plates were then centrifuged at 1000* g* for 3 min and 150 μl of the supernatant was transferred to flat bottom 96-well plates and spun again to remove air bubbles. Absorbance was measured using a SPARK multifunctional microplate reader (TECAN) at 415 nm. LogEC_50_ values were determined by fitting the data to 4-parameter sigmoidal dose–response curves using Prism (GraphPad).

### Hydration status of RBCs over time

Washed RBCs were pre-cultured for 24 h in our standard culture conditions in culture dishes in RPMI/albumax at 5% haematocrit in incubation chambers flushed with a gas mixture of 5% CO_2_, 5% O_2_, 90% N_2_ and gentle shaking to keep cells in suspension. Then 1.5 ml of these cultures were treated with the indicated concentrations of Yoda1 or Jedi2 or corresponding volumes of DMSO as control. Cultures were transferred to 35 mm cell culture dishes and cultured as described above for up to 24 h. At the indicated time points, 250 µL of suspension was used to determine the hydration status by osmotic fragility assays.

### Video microscopy

For the observation of the cell shape, RBCs were washed and re-suspended in complete medium and settled in a poly-L lysine (PLL) coated slide chamber (Ibidi µ-slide VI). Cells were washed thrice with complete medium to remove non-adherent cells and then flushed with complete medium containing either Yoda1 (1 µM or 5 µM) or DMSO and time–lapse video microscopy recorded with 63 × magnifications at a speed of 1 image/2 s. To observe the recovery of cell shape, RBCs in complete medium were treated with 10 µM Yoda1 (at this concentration, all RBCs become strong echinocytes), placed in glass bottom dishes (MatTek), and time–lapse video microscopy recorded with 63 × magnifications over 2 ½ h at 6 images/min. For the observation of egress and invasion events, late-stage parasites were purified on VarioMACS columns, washed and re-suspended in complete medium and incubated for up to 5 h in the presence of 1.5 µM PKG inhibitor C2 to block egress. For egress studies, parasites were washed twice and re-suspended in complete medium containing 5 µM Yoda1. For control samples 0.5% DMSO was used. 100 µl of each cell suspension was loaded into adjacent wells (Ibidi µ-8 well slide) and recorded in alternation. For invasion, washed parasites were mixed with RBCs that had been pre-treated with 5 µM Yoda1 or 0.5% DMSO for 10 min in RPMI, washed three times and incubated at 37 °C for 5 h. Samples were loaded in glass bottom dishes (MatTek). All events were imaged using an inverted brightfield microscope (Axioobserver, Zeiss), equipped with an incubation chamber set at 37 °C and 5% CO_2_ and a CoolSNAP HQ2 CCD camera (Photometrics). Time-lapse experiments were performed, by automatic acquisition of designated fields using a 63 × Apochromat objective (NA 1.4). Image treatment and analysis were performed using Zen software (Zeiss).

### Scanning electron microscopy

For environmental scanning electron microscopy (ESEM), samples were fixed at room temperature with 2.5% glutaraldehyde in 0.1 M cacodylate buffer followed by 1% osmium tetroxide, and washed in water. A 10 µl drop of suspension was loaded on the sample carrier and imaged in a FEI Quanta200 FEG microscope in ESEM mode using the gaseous secondary electron detector. The stage was set-up at 2 °C, the acceleration voltage was 15 kV and the working distance 10 mm. Water was then progressively removed by cycles of decreasing pressure/injection of water, until reaching equilibrium at the dew point. The minimal final pressure in the chamber was 350 Pa. Pictures were taken with a dwell time of 6 µs.

### Generation of RBCs with altered Na^+^/K^+^ content

To generate RBCs with a cellular Na^+^ and K^+^ content similar to the situation observed upon treatment with Yoda1, RBCs were incubated on ice in the presence of 40 µg/ml of the ionophore nystatin (Sigma–Aldrich) in a saline solution of either 75 mM NaCl/75 mM KCl or 110 mM NaCl/40 mM KCl in 5 mM Hepes pH 7.4 and 55 mM sucrose. Upon incubation during 20 min, cells were collected by centrifugation and washed twice for 20 min on ice with the identical solution containing 0.1% BSA to efficiently remove nystatin. The cellular Na^+^/K^+^ content was measured by spectroscopy with a Solaar spectrometer immediately after the washing and after an additional incubation of the RBCs for 90 min at 37 °C in RPMI.

### Measurements of sodium, potassium and water

Fresh venous blood was washed three times at room temperature in RPMI and set at 30% haematocrit. Three aliquots of 1.5 ml were treated with 1 µM Yoda1 or DMSO (control) for 10 min at room temperature, then rinsed three times in RPMI. For Na^+^ and K^+^ content measurements, 500 µl of cell suspension were taken to fill 3 nylon tubes that were centrifuged for 10 min at 4 °C, 20,000* g* at the end of Yoda1 washing (*t* = 0) and up to 24 h incubation at 37 °C and 5% CO_2_ in complete medium. The pellet of RBCs was extracted and weighed immediately. Dry weight was measured after overnight heating (80 °C). Water content was calculated with a correction of 3.64% corresponding to trapped medium between packed cells. Intracellular ions were extracted from dried pellets by overnight incubation at 4 °C in 5 ml milliRho water (Millipore). Na^+^ and K^+^ were measured by flame spectroscopy with a Solaar spectrometer.

### RBC attachment assays

Merozoite attachment to RBCs was quantified in the presence of the inhibitor of actin polymerisation cytochalasin D (cytD) following the protocol of Paul et al. [33]. RBCs were washed twice in RPMI and treated with either 5 µM Yoda1 or DMSO vehicle for 10 min at RT. Preparations were then washed three times in RPMI and eventually re-suspended in complete medium (RPMI, 0.5% albumax) and cultured for 4–5 h in standard conditions. Schizont- and segmenter-infected RBCs were purified from highly synchronised parasite cultures by magnetic purification on MACS columns (Milteny), efficiently removing uninfected RBCs. Purified infected RBCs were cultured at 37 °C for 4–5 h in complete medium in the presence of 1.5 µM C2 to allow parasite maturation while blocking egress. C2 was removed by centrifuging and re-suspending cells in an equal volume of fresh complete medium. 100 µL of cell suspension were immediately added to 2 ml tubes containing 100 µL of Yoda1 or DMSO pre-treated RBC suspensions in the presence of 1 µM cytD (allowing attachment but inhibiting invasion), 200 µg/ml heparin (inhibiting attachment and invasion) or 0.1% DMSO vehicle (allowing invasion). Assays were done in triplicates at a final haematocrit of 2.5%. Tubes were gazed for 3 s with 5% O_2_, 5% CO_2_ and incubated for 3 h at 37 °C on a spinning wheel for mixing. Cells were fixed by adding to each tube 800 µL of a sucrose-stabilised solution (0.116 M) in PBS supplemented with 2% glutaraldehyde [32]. The starting parasitemia (3–6%) was determined by fixing cells immediately after addition of iRBCs. Samples were stored at 4 °C until later processing.

For quantification of attachment by FACS, 10^7^ cells (200 µL fixed suspension) were transferred to microtubes, pelleted at 300 g for 90 s and blocked with 300 µL of a sucrose-stabilised solution (0.116 M) in PBS supplemented with 0.1 M glycine for 30 min at RT. Cells were washed twice with 500 µL PBS/sucrose and then incubated 30 min with 3.3 × SYBR Green (Thermo Fisher Scientific). DNA dye was removed by a simple wash in PBS/sucrose and samples analysed on a FACS-CANTO II cytometer (BD Biosciences) using processed uninfected RBCs as negative control. RBCs with attached merozoites (in the presence of cytD) are detected with a fluorescence signal identical to ring stage iRBCs (in the absence of cytD and heparin) while heparin treatment prevents attachment and invasion. Data were expressed as percentage of DMSO-treated control RBCs after subtraction of baseline measurements of parasitemia in the presence of heparin.

### Calcium measurements

Assays were either preformed in saline solution (145 mM NaCl, 4 mM KCl, 0.15 mM MgCl_2_, 10 mM glucose, 10 mM HEPES, 2 mM CaCl_2_) or in RPMI 1640 medium without phenol red (Gibco # 11835063) supplemented with 10 mM HEPES. RBCs were washed three times in the respective medium. 5 × 10^7^ RBCs were labelled with 5 µM Fluo4-AM and incubated for 30 min at 37 °C. Cells were washed three times, re-suspended in the respective medium and incubated for de-esterification for 30 min at room temperature. Suspensions of 1 × 10^6^ RBCs in 100 µL were distributed in 96-well plates. Baseline fluorescence measurements were taken over 2 min at 20 s intervals in a SPARK multifunctional microplate reader (TECAN) with excitation at 485 nm and emission at 535 nm. Assays were then started by adding 100 µL of double concentrated drugs in their respective medium and mixed using a multichannel pipette. Measurements were followed over 15–30 min. Fluorescent signals were calculated after subtraction of values of mock-treated samples.

### Calcium measurements by video microscopy

RBCs were washed twice in RPMI and labelled for 30 min with 5 µM Fluo4-AM at 37 °C. After three washes with RPMI cells were re-suspended in complete medium and allowed to de-esterify at room temperature for 30 min. During this time cells were allowed to settle in poly-L lysine (PLL) coated slide chambers (Ibidi µ-slide VI). Non-adherent cells were removed by repeated washes. Alternate cycles of acquisitions every 4 s of brightfield and fluorescent signals were started before the addition of complete medium containing 5 µM Yoda1, 1 mM Jedi2 or DMSO vehicle and followed over about 5 min. Using Fiji software [63] relative fluorescent signals for defined areas of identical size covering individual RBCs were obtained. Background fluorescence was obtained by calculating the mean of five identical areas without cells and subtracted from all values.

### Statistics and data analysis

Data analysis and plots were generated using Excel (Microsoft) and Prism (GraphPad). Osmotic fragility curves were generated by fitting the data to a four-parameter sigmoidal dose–response curve and LogEC_50_ values were calculated. Statistical significance was determined by non-parametric Mann–Whitney test for *n* > 3, or by unpaired two-tailed *t*-test (GraphPad Prism). Means associated with the standard error of the mean (SEM) are illustrated on the figures.

## Supplementary Information

Below is the link to the electronic supplementary material.

Supplementary file1 (PDF 218 KB)

Supplementary file2 (AVI 7290 KB)

Supplementary file3 (AVI 5758 KB)

Supplementary file4 (AVI 4797 KB)

Supplementary file5 (AVI 5701 KB)

Supplementary file6 (AVI 5651 KB)

Supplementary file7 (AVI 4501 KB)

## Data Availability

All data generated or analyzed during this study are included in this published article and its supplementary information files.

## Abbreviations

GOF: Gain-of-function
Hb: Haemoglobin
PKG: Protein kinase G
RBC: Red blood cell
SCD: Sickle cell disease
