# Supplementary material for: Pharmacological activation of PIEZO1 in human red blood cells prevents Plasmodium falciparum invasion
Source: Cell Mol Life Sci. 2023 Apr 18;80(5):124. doi: 10.1007/s00018-023-04773-0 (PMC10113305; doi:10.1007/s00018-023-04773-0)
Supplement: Supplementary file 1 — Supplementary file1 (PDF 218 KB) [file 18_2023_4773_MOESM1_ESM.pdf]

Supplemental Data

Figures

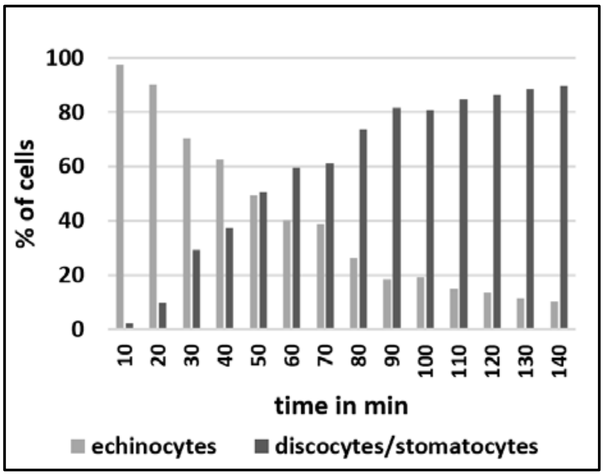

Supplemental Fig. 1: RBC shape changes over prolonged application (2½ hours) of Yoda1. Graphical representation of cell shape changes over time (data extracted from supplemental Video 4).

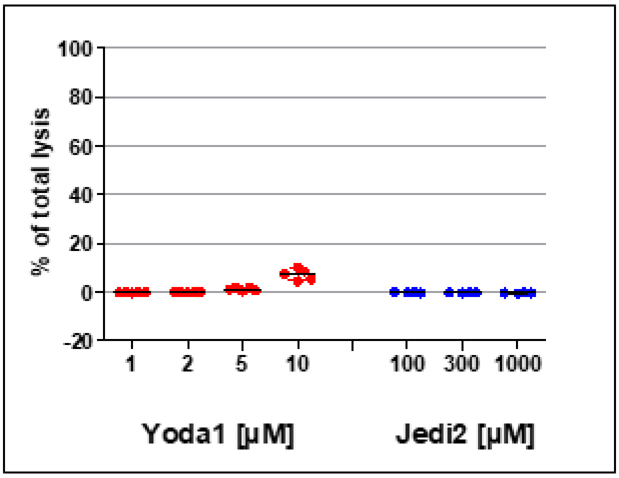

Supplemental Fig. 2: Haemolytic activities of the PIEZO1 activators Yoda1 and Jedi2. Blood of different donors was cultured for 24 hours at 2.5% haematocrit in standard culture conditions (RPMI without phenol red and with 0.5% albumax) in the presence of the indicated concentrations of compounds. Haemoglobin released into the culture supernatant was quantified by measuring the absorbance at 540 nm in a TECAN spectrophotometer and expressed as percentage of total lysis induced by the addition of 0.15% saponin. Shown are mean and SD, n = 5 for Yoda1 and n = 4 for Jedi2, all performed in triplicates.

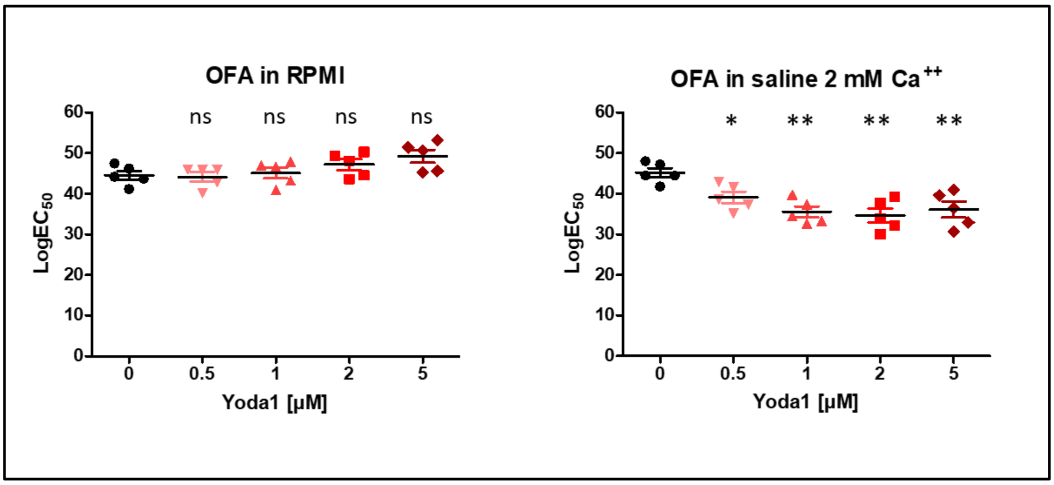

Supplemental Fig. 3: Osmotic fragility assays after incubation in RPMI medium (left panel) and saline solution containing 2 mM $CaCl_2$ (right panel). Blood of five different donors was incubated with the indicated concentrations of Yoda1 for 30 minutes either in saline solution (149 mM NaCl, 4 mM KCl, 2 mM $CaCl_2$, 2 mM Hepes, pH 7.4) or in RPMI medium before determining their osmotic fragility after 5 min in varying iso- and hypo-osmotic solutions. Shown are the mean and SEM, n = 5, all performed in duplicates. * = $p<0.05$, ** = $p<0.01$, ns = not significant (Mann-Whitney test).

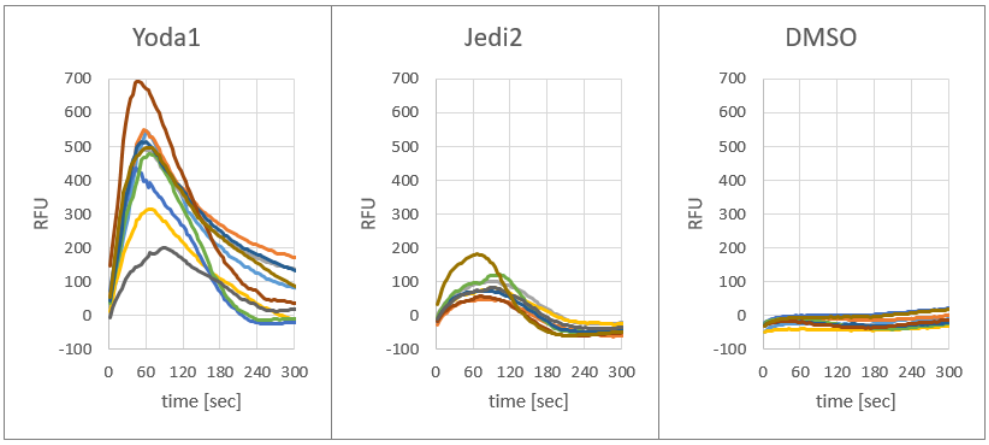

Supplemental Fig. 4: Fluorescence readings of Fluo4-AM loaded RBCs by video microscopy. Each line represents the reading of an individual cell over time. Time = 0 corresponds to the addition of complete medium RPMI/albumax containing 5 µM Yoda1, 1 mM Jedi2 or DMSO vehicle and followed over 5 min.

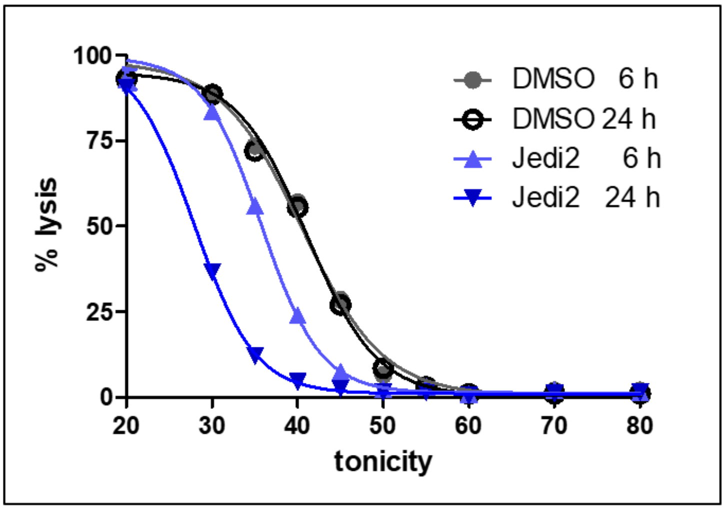

Supplemental Fig. 5: Osmotic fragility curves of human RBCs after prolonged incubations with 1 mM Jedi2 or DMSO solvent in RPMI/albumax medium. Shown is one representative example done in duplicates (n=3).

Videos

Video 1

Absence of changes of the RBC cell shape upon the addition of DMSO vehicle. Human RBCs were loaded in complete medium RPMI/albumax in poly-L-lysine treated 15 µ-Slide VI (ibidi) and imaged using a 63× Apochromat objective (NA 1.4) on an inverted brightfield microscope (Axioobserver, Zeiss), equipped with an incubation chamber set at 37°C and 5% CO_2 and a CoolSNAP HQ2 CCD camera (Photometrics). Time-lapse acquisition over 20 minutes at 1 image every 10 seconds was started prior to the addition of DMSO vehicle (defined as time = 0) in complete medium.

Video 2

Changes of the RBC cell shape upon the addition of 1 µM Yoda1. Human RBCs were loaded in complete medium RPMI/albumax in poly-L-lysine treated 15 µ-Slide VI (ibidi) and imaged using 63× magnification. Time-lapse acquisition over 20 minutes (1 image every 10 seconds) was started prior to the addition of 1 µM Yoda1 (defined as time = 0) in complete medium.

Video 3

Changes of the RBC cell shape upon the addition of 5 µM Yoda1. Human RBCs were loaded in complete medium RPMI/albumax in poly-L-lysine treated 15 µ-Slide VI (ibidi) and imaged using 63× magnification. Time-lapse acquisition (1 image every 2 seconds) was started prior to the addition of 5 µM Yoda1 in complete medium (defined as time = 0). Note the reduced duration of the recording (70 sec) compared to 20 min for 1 µM Yoda1 and DMSO control.

Video 4

To observe the recovery of RBC cell shape, RBCs in complete medium were treated with 10 µM Yoda1 (at this concentration, all RBCs become strong echinocytes), directly placed in glass bottom dishes (MatTek), and time–lapse video microscopy recorded at 37°C and 5% CO_2 with 63 x magnifications over 2 ½ h. Time indicated in min. Scale bar 10 µM. A graphical representation of cell shape changes over time is presented in Supplemental Fig. 1.

Video 5

Representative video of invasion events in complete medium with mock-treated RBCs. RBCs that become invaded are labelled 1 and 2. Selected images of RBC 1 are shown in Figure 4B. Scale bar 5 µM.

Video 6

Representative video of the reaction of RBCs pre-treated with 5 µM Yoda1 to contact with *P. falciparum* merozoites and absence of invasion. Scale bar 2 µM.
